# Alterations in the Transcriptome of Rye Plants following the *Microdochium nivale* Infection: Identification of Resistance/Susceptibility-Related Reactions Based on RNA-Seq Analysis

**DOI:** 10.3390/plants10122723

**Published:** 2021-12-10

**Authors:** Ivan Tsers, Azat Meshcherov, Olga Gogoleva, Olga Petrova, Natalia Gogoleva, Mira Ponomareva, Yuri Gogolev, Viktor Korzun, Vladimir Gorshkov

**Affiliations:** 1 Federal Research Center Kazan Scientific Center of the Russian Academy of Sciences, 420111 Kazan, Russia; ivantsers@gmail.com (I.T.); strosaz125@gmail.com (A.M.); gogolewaoa@yandex.ru (O.G.); poe60@mail.ru (O.P.); negogoleva@gmail.com (N.G.); smponomarev@yandex.ru (M.P.); gogolev.yuri@gmail.com (Y.G.); viktor.korzun@kws.com (V.K.); 2 Kazan Institute of Biochemistry and Biophysics, Federal Research Center “Kazan Scientific Center of the Russian Academy of Sciences”, 420111 Kazan, Russia; 3 KWS SAAT SE & Co. KGaA, Grimsehlstr. 31, 37555 Einbeck, Germany

**Keywords:** *Microdochium nivale*, plant resistance, plant susceptibility, plant–microbe interactions, RNA-Seq analysis, rye

## Abstract

*Microdochium nivale* is a progressive and devastating phytopathogen that causes different types of cereal crop and grass diseases that are poorly characterized at the molecular level. Although rye (*Secale cereale* L.) is one of the most resistant crops to most of the phytopathogens, it is severely damaged by *M. nivale*. The recent high-quality chromosome-scale assembly of rye genome has improved whole-genome studies of this crop. In the present work, the first transcriptome study of the *M. nivale*-infected crop plant (rye) with the detailed functional gene classification was carried out, along with the physiological verification of the RNA-Seq data. The results revealed plant reactions that contributed to their resistance or susceptibility to *M. nivale*. Phytohormone abscisic acid was shown to promote plant tolerance to *M. nivale*. Flavonoids were proposed to contribute to plant resistance to this pathogen. The upregulation of plant lipase encoding genes and the induction of lipase activity in *M. nivale*-infected plants revealed in our study were presumed to play an important role in plant susceptibility to the studied phytopathogen. Our work disclosed important aspects of plant-*M. nivale* interactions, outlined the directions for future studies on poorly characterized plant diseases caused by this phytopathogen, and provided new opportunities to improve cereals breeding and food security strategies.

## 1. Introduction

*Microdochium nivale* (Fr.) Samuels and I.C. Hallett is a phytopathogenic psychrotolerant fungus able to cause different types of plant diseases (pink snow mold, foot rot, leaf blight, and head blight) throughout the entire growing season, including winter, when it thrives on plants overwintering under the snow cover [1,2,3,4,5]. *M. nivale*-caused diseases are associated with plant tissue desiccation, coupled with the extensive growth of white or pink mycelium and the formation of orange sporodochia. In addition, this pathogen leads to the reduction of seed germination, as well as the pre- and post-emergence death of seedlings. Yield damage from *M. nivale* infection ranges from 20% to total loss of the cereal crops [6,7].

No plant cultivars with full resistance to *M. nivale* exist, and most cultivars are highly susceptible to this pathogen [8]. Therefore, *M. nivale* is a serious problem for the breeding and cultivation of winter cereals (rye, wheat, oat, barley, triticale), as well as forage and turf grasses [9,10]. In addition, the molecular and physiological criteria of *M. nivale* infection are poorly investigated and form additional obstacles in disease management.

It has been shown that *M. nivale* infections are associated with the accumulation of phenolic compounds [11] and hydrogen peroxide [12], as well as callose deposition [13]. The concentration of several amino acids (glutamine, arginine, phenylalanine, and asparagine) increases in response to *M. nivale* infection [14]. Some physiological reactions following *M. nivale* infection have been demonstrated to be host plant genotype-dependent. Superoxide dismutase (SOD) activity was decreased in triticale during *M. nivale* infection [15]; herewith, changes in SOD activity varied in different host plant cultivars [16]. The level of lipid peroxidation in *M. nivale*-infected triticale cultivars has been shown to correspond to the level of host susceptibility: the more susceptible the cultivar, the more pronounced the lipid peroxidation [15].

Several plant physiological traits have been proposed to be associated with the increased resistance to *M. nivale*. Plant cold acclimation (hardening) leading to frost tolerance is considered to provide increased resistance to *M. nivale* [17,18,19] as well as to other phytopathogens [20,21]. The hardening-induced increase in resistance to *M. nivale* can be expressed to different degrees depending on plant genotypes [2]. During cold acclimation, total soluble carbohydrates (TSC), including high-molecular-weight fructans, are accumulated in plant tissues. Herewith, rye or annual bluegrass cultivars that accumulated more TSC/fructans during cold acclimation are more resistant to *M. nivale* infection than the cultivars that accumulated less TSC/fructans [14].

The level of *M. nivale*-induced activation of peroxidase activity has been shown to correspond to the level of resistance of *Lolium* spp. and *Festuca* spp. plants to this pathogen [16]. It has also been found that the pre-infectional content of salicylic acid (SA) and phenolic compounds were lower, whereas the activity of phenylalanine ammonia-lyase and abscisic acid (ABA) content was higher in more resistant to *M. nivale Festulolium* cultivars than in more susceptible ones [22]. However, the correlation between ABA content and resistance to *M. nivale* has not been revealed in rye plants [23]. The treatment of wheat plants with ABA or cold acclimation induced the expression of genes encoding fungistatic proteins that repressed the in vitro growth of phytopathogens, including *M. nivale* [24,25]. However, the influence of these proteins on *M. nivale* growth within the host plant has not been defined as well as the role of ABA in plant-*M. nivale* interactions.

Despite several *M. nivale*-induced plant reactions having been described, the general picture of plant metabolism modulation during *M. nivale* infection has not been ascertained. OMICs, including transcriptome profiling, enable predicting the physiological alterations in an organism at the whole-genome level, and thus allow tracking the reactions which are the most crucial for the target biological process [26,27]. For plant-microbial pathosystems, the modulation of plant gene expression often reflects the defense and susceptible responses. Defense responses provide the restriction (at least to some extent) of pathogen propagation [28]. In contrast, susceptible responses driven by pathogen manipulation promote pathogen fitness in planta, and exacerbate disease development [29]. The identification of defense and susceptible responses is a key for understanding the crucial aspects of plant–pathogen interaction, and for disease management.

The only OMICs study performed to date on *M. nivale*-infected plants—transcriptome profiling of perennial ryegrass (*Lolium perenne* L.)—was accentuated on genes triggered by pathogen-associated molecular patterns (PAMP) [30]. The transcriptome alterations following the *M. nivale* infection have not been analyzed for any of the crop plants. Although rye (*Secale cereale* L.) is one of the most resistant crops to many phytopathogens, it is severely damaged by *M. nivale* [8,31,32]. Until recently, genomic (transcriptomic) studies were a challenge for rye plants due to the lack of a well-assembled and well-annotated genome. However, in 2021, a high-quality chromosome-scale reference genome of rye was assembled and annotated [33]. This has opened up the possibility for genome-wide studies of this important global food crop, including the investigations of pathological processes.

Our study aimed to predict the rye plant responses modulated following the *M. nivale* infection based on transcriptome profiling, and to perform the physiological verification of the RNA-Seq data by biochemical assays or treatment tests. Our investigation was directed towards searching the defense and susceptible responses of rye plants to *M. nivale* infection.

## 2. Results

### 2.1. Modulation of Gene Expression in Rye Plants Following M. nivale Infection

The mRNA-libraries corresponding to the control and *M. nivale*-infected rye plants were sequenced in three biological replicates yielding 19–29 million reads aligned to reference rye transcripts per replicate (Supplementary Table S1). In total, 20,554 rye plant genes were considered to be expressed in our study. Among them, 1008 genes were expressed differentially in *M. nivale*-infected plants compared to the control plants: 745 genes were up- and 263 were downregulated during the infection. Differentially expressed genes (DEGs) were split into different categories and subcategories (Supplementary Tables S2 and S3) according to their classification (eggNOG, KEGG, and CAZy) and annotation (UniProt) in different databases. Some gene categories and subcategories assembled in our study based on merging the information from different sources are described below.

#### 2.1.1. The Cell Wall Category

34 DEGs (19 up- and 15 downregulated) encode plant cell wall proteins/enzymes. The most pronounced upregulation in the cell wall category was observed for genes encoding chitinase-like proteins: 7 genes (SECCEUnv1G0532050.1, SECCEUnv1G0532070.1, SECCE3Rv1G0149460.1, SECCE3Rv1G0149470.1, SECCE7Rv1G0498520.1, SECCE3Rv1G0166920.1, SECCE2Rv1G0142590.1) were upregulated during the infection with log_2_FC values up to 10 for one of them (SECCEUnv1G0532050.1). Cellulose-related genes were not found among DEGs in our study. Seven genes related to the metabolism of cross-linking glycans (CLGs) were DEGs. Among them, 2 CLG-biosynthetic genes (encoding cellulose-synthase-like proteins (CSLs)) were upregulated (SECCE2Rv1G0103270.1, SECCE3Rv1G0143430.1), while 4 of 5 CLG-degradation/modification-related DEGs (1 DEG encoding endoglucanase [SECCE1Rv1G0013110.1], and 3 DEGs encoding xyloglucan endotransglycosylase/hydrolase, XTH [SECCE5Rv1G0371330.1, SECCE7Rv1G0507960.1, SECCE7Rv1G0508040.1]) were downregulated. The only upregulated CLG-degradation-related gene was the one encoding xylanase (SECCE7Rv1G0470630.1). Pectin biosynthetic genes were non-differentially expressed (non-DEGs) in our study, while 4 polygalacturonan-degradation-related genes were DEGs: 2 of them (encoding pectate-lyase-like protein [SECCE1Rv1G0051920.1] and pectin methylesterase inhibitor-like protein [SECCE2Rv1G0121130.1]) were upregulated, and 2 (encoding pectinesterase-like protein [SECCE2Rv1G0073490.1] and pectinesterase inhibitor-like protein [SECCE7Rv1G0505350.1]) were downregulated. As for the lignin polymerization-related genes, 2 DEGs encoding laccases (SECCE3Rv1G0210800.1, SECCE1Rv1G0006560.1) were strongly upregulated during the infection, while 3 DEGs encoding dirigent-like proteins (SECCE3Rv1G0157150.1, SECCE7Rv1G0463220.1, SECCE7Rv1G0522440.1) that contribute to lignan biosynthesis [34] were downregulated.

#### 2.1.2. The Primary Metabolism Category

**Figure 1:**
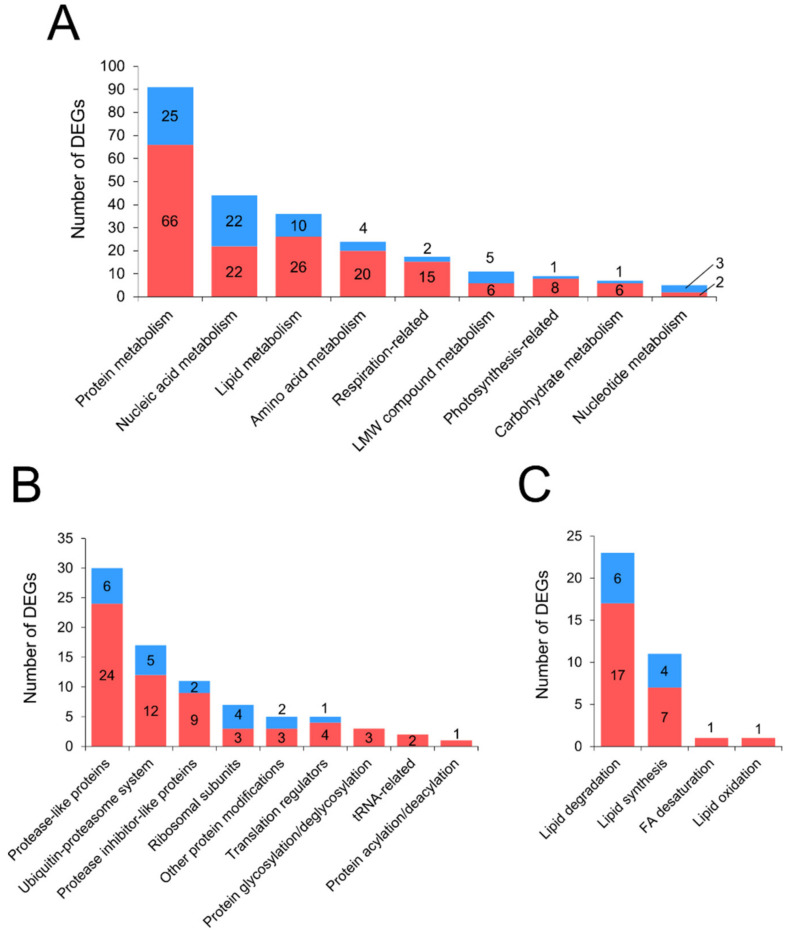
The number of up- (red) and downregulated (blue) differentially expressed genes (DEGs) attributed to the ‘Primary metabolism’ category in rye plants infected with *Microdochium nivale*. (**A**) The number of DEGs in the ‘Primary metabolism’ category. (**B**) The number of DEGs in the ‘Protein metabolism’ subcategory. (**C**) The number of DEGs in the ‘Lipid metabolism’ subcategory. LMW, low molecular weight; FA, fatty acid.

Overall, 244 DEGs (171 upregulated and 73 downregulated) were attributed to the “Primary metabolism” category. The majority of upregulated DEGs of this category were distributed between 4 subcategories: “Protein metabolism”, “Nucleic acid metabolism”, “Lipid metabolism”, “Amino acid metabolism” (Figure 1A). Many respiration-related genes (predominantly those that are associated with the electron transport chain) were upregulated, which is rather typical of the biotic stress [35].

Three major sub-subcategories of the “Protein metabolism” subcategory were comprised of mostly upregulated DEGs encoding (1) protease-like proteins (e.g., serine/cysteine proteases, carboxypeptidases, metalloendoproteases), (2) proteins related to the proteasome-dependent protein degradation (e.g., ubiquitin, ubiquitin-conjugating, and hydrolyzing enzymes), and (3) protease inhibitors (e.g., serpins, wound-induced protease inhibitors) (Figure 1B). The majority of DEGs related to the “Lipid metabolism” subcategory were attributed to the “Lipid degradation” sub-sub category (Figure 1C). Two main groups of upregulated DEGs in the “Lipid degradation” sub-sub category were (1) lipase-like proteins (including patatins) and (2) esterase-like proteins with uncertain activity potentially related to lipids (GDSL esterase/lipase, esterase/lipase/thioesterase family proteins). Within the “Amino acid metabolism” subcategory, 8 of 20 upregulated DEGs were related to arginine metabolism, which has been previously shown to be enhanced during different plant–pathogen interactions [15,36,37].

#### 2.1.3. The Secondary Metabolism Category

Moreover, 41 (31 up- and 10 downregulated) DEGs encoding the enzymes involved in the biosynthesis of secondary metabolites were revealed. The most pronounced upregulation in this gene category was observed for terpenoid-related genes. Eleven DEGs encoding enzymes of terpenoid synthesis (mostly those related to non-mevalonate pathway) were strongly upregulated, indicating that the production of these compounds might be enhanced during *M. nivale* infection. Five alkaloid metabolism-related genes were also upregulated. Seven DEGs related to flavonoid biosynthesis were revealed. However, flavonoid-biosynthetic DEGs were almost equally distributed among up- (4 genes) and down- (3 genes) regulated genes, and, therefore, it was hard to judge whether flavonoid synthesis was enhanced or repressed in the *M. nivale*-infected plants. Six DEGs encoding phenylalanine ammonia-lyase (PAL) (SECCE2Rv1G0112880.1, SECCE2Rv1G0112860.1 SECCE6Rv1G0399260.1, SECCE2Rv1G0086880.1, SECCE2Rv1G0112840.1, SECCE2Rv1G0086850.1) and 1 DEG for isochorismate synthase 2 (ICS2) (SECCE5Rv1G0324680.1) were upregulated indicating that the initial reactions of phenylpropanoid biosynthesis might be enhanced during *M. nivale*-caused infection. We also revealed two upregulated DEGs encoding 4-hydroxy-7-methoxy-3-oxo-3,4-dihydro-2H-1,4-benzoxazin-2-yl-glucoside- beta-D-glucosidase (SECCE5Rv1G0338300.1, SECCE2Rv1G0113270.1). The reaction catalyzed by this enzyme yields DIMBOA, a powerful *Poaceae*-specific natural antibiotic that is produced in response to insect attack [38].

#### 2.1.4. The Stress-Related Category

In total, 78 DEGs (57 upregulated and 21 downregulated) were attributed to the “Stress-related” category. The most pronounced upregulation was observed for 16 DEGs encoding xenobiotic detoxification-related proteins such as glutathione-S-transferases and MATE efflux-like proteins. We also revealed several strongly upregulated DEGs encoding pathogenesis-related (PR) proteins: 2 DEGs for PR-1 (SECCE7Rv1G0480890.1, SECCE5Rv1G0308920.1), 1 DEG for PR-5-like (thaumatin) (SECCEUnv1G0561830.1), 1 DEG for PR-4-like (SECCE6Rv1G0436660.1), 2 DEGs for putative PR-5-like proteins (SECCE2Rv1G0142260.1, SECCE2Rv1G0142220.1). Eleven upregulated DEGs encode proteins involved in the perception of pathogen elicitors and the transduction of the corresponding signal: R-like proteins of the NBS-LRR class and RIN-interacting-like kinases (RIPK-like). Seven DEGs related to the metabolism of the reactive oxygen species (ROS) were upregulated: 4 DEGs for peroxidases (SECCE3Rv1G0191290.1, SECCE3Rv1G0191300.1, SECCE5Rv1G0354500.1, SECCE3Rv1G0193060.1), 2 DEGs encoding the enzymes involved in the synthesis of ascorbate (L-gulonolactone oxidase [SECCE7Rv1G0493060.1] and monodehydroascorbate reductase [SECCE7Rv1G0484880.1]), and one DEG for thioredoxin-like protein (SECCE3Rv1G0206530.1).

#### 2.1.5. The Signaling-Related Category

Furthermore, 197 DEGs (161 upregulated and 36 downregulated) were attributed to the signaling-related category. The “Phytohormone-related”, “Receptor-like kinases”, and “Transcription factors” subcategories were most enriched with upregulated DEGs.

In addition, 23 up- and 3 downregulated DEGs encode the proteins related to phytohormone metabolism and signaling. Genes encoding some enzymes of jasmonic acid (JA)-biosynthetic branch (13-S lipoxygenase-like protein [SECCE4Rv1G0244470.1] and peroxisomal 12-oxophytodienoate reductase [SECCE5Rv1G0371440.1]) were upregulated following *M. nivale* infection, while genes for other enzymes of this branch (allene oxide synthase and allene oxide cyclase) were non-DEGs. Two genes, whose products are involved in JA-signaling (MYB21-like protein [SECCE1Rv1G0058750.1] and JAZ-like protein [SECCE7Rv1G0464250.1]) were upregulated. Two ethylene-biosynthetic genes encoding 1-aminocyclopropane-1-carboxylate synthase (ACS) (SECCE2Rv1G0115020.1, SECCE7Rv1G0508560.1) were upregulated as well as one EIN3 gene (SECCE3Rv1G0202840.1), whose product is involved in ethylene signaling. Genes encoding phenylalanine ammonia-lyase (PAL) (SECCE2Rv1G0112880.1, SECCE2Rv1G0112860.1 SECCE6Rv1G0399260.1, SECCE2Rv1G0086880.1, SECCE2Rv1G0112840.1, SEC-CE2Rv1G0086850.1) and isochorismate synthase 2 (ICS2) (SECCE5Rv1G0324680.1) responsible for salicylic acid (SA) biosynthesis were strongly upregulated. However, the products of catalytic actions of PAL and ICS2 are precursors of not only SA but also a wide range of phenylpropanoids (including monolignols). Therefore, it is hard to speculate whether the SA synthesis was enhanced during *M. nivale* infection. Given that genes encoding WRKY51 (SECCE3Rv1G0184290.1, SECCE1Rv1G0051050.1), a transcription factor shown to be positively regulated by SA and involved in the repression of JA responses [39], was upregulated it may be presumed that SA might play some role in plant-*M. nivale* interaction.

**Figure 2:**
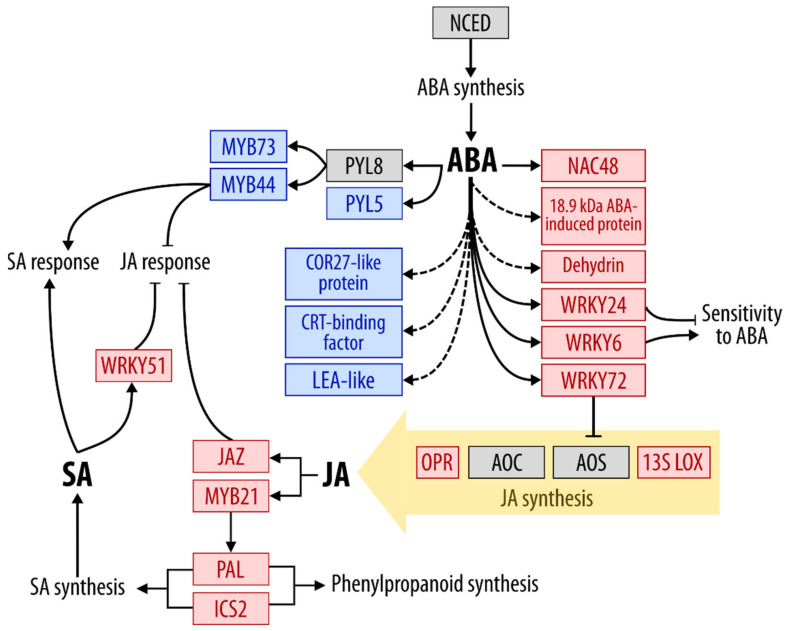
The expression pattern of genes related to abscisic acid (ABA), jasmonic acid (JA), and salicylic acid (SA) metabolism and signaling in *M. nivale*-infected rye plants. Red and blue boxes show proteins for which at least one DEG was up- or downregulated, respectively. Grey boxes show proteins encoded by non-differentially expressed genes. The known regulation is shown as solid lines, while the predicted regulation—as dashed lines. Positive regulation is shown as a line ending with arrowhead, negative regulation—as a line ending with the perpendicular endpoint. Abbreviations: 13S-LOX, 13S-lipoxygenase; ABA, abscisic acid; AOC, allene oxide cyclase; AOS, allene oxide synthase; COR27, cold-regulated 27; CRT, C-repeat element; ICS2, isochorismate synthase 2; JAZ, jasmonate-zim domain; NCED, 9-cis-epoxycarotenoid dioxygenase. OPR, 12-oxophytodienoic acid reductase; PAL, phenylalanine-ammonia lyase; PYL5/8, pyrabactin-resistant-like 5/8. The details are discussed in the text.

Although no DEGs encoding abscisic acid (ABA) biosynthetic enzymes were revealed in our study, the expression of many ABA-regulated genes differed in control and *M. nivale*-infected plants. Some ABA regulated genes were downregulated: 4 DEGs encoding proteins similar to CBF (C-repeat binding factors, also known as Dehydration Response Element Binding factors, DREB) (SECCE5Rv1G0340350.1, SECCE5Rv1G0340450.1, SECCE5Rv1G0340490.1, SECCE5Rv1G0340480.1); 3 DEGs for COR27-like protein (Cold-regulated 27) (SECCE6Rv1G0403060.1, SECCE2Rv1G0082180.1, SECCE7Rv1G0464580.1); 2 DEGs encoding for late embryogenesis abundant (LEA)-like protein (SECCE3Rv1G0171230.1, SECCE3Rv1G0168390.1); 1 DEG for ABA receptor Pyrabactin-Resistant-like 5 (PYL5) (SECCE7Rv1G0480690.1); and DEGs encoding MYB73-like (SECCE1Rv1G0033420.1) and MYB44-like proteins (SECCE6Rv1G0451850.1) (Figure 2). CBF/DREB and *AtCOR27* genes have been previously shown to be regulated by ABA [40,41]. MYB73 and MYB44 have been demonstrated to act as ABA-regulated transcription factors [42]; herewith, AtMYB44 represses JA-regulated responses and enhances SA-regulated ones via interaction with AtWRKY70 [43].

Simultaneously, some ABA-regulated genes were upregulated in *M. nivale*-infected plants, including genes for transcription factors WRKY (WRKY6, 24, and 72 [SECCE3Rv1G0170470.1, SECCE3Rv1G0196160.1, SECCE3Rv1G0161830.1]) and NAC48 (also known as OsNAC6) (SECCE3Rv1G0204240.1) (Figure 2). WRKY6 has been shown to regulate the sensitivity to ABA in *A. thaliana* plants [44], while WRKY24 acts as a negative regulator for ABA sensitivity [45], and ABA-regulated transcription factor WRKY72 represses the expression of allene oxide synthase gene (that was downregulated in our study) thus impairing the JA biosynthesis [46]. NAC48 has been demonstrated to be positively regulated by ABA and involved in drought tolerance [47]. In addition, the levels of SECCE4Rv1G0295640 and SECCE6Rv1G0415480.1 gene transcripts were strongly increased (log_2_FC ≈ 11 and 8, respectively) in *M. nivale*-infected plants. The first gene encodes 18.9 kDa ABA-induced protein [48], and the second one encodes dehydrin-like protein—an LEA protein, whose production is activated by ABA [49]. Thus, our data indicate that the reprogramming of expression of ABA-responsive genes took place during *M. nivale* infection.

In addition to phytohormone-regulated DEGs, 52 up- and 5 downregulated DEGs encoding receptor-like kinases were revealed in the “Signaling-related” gene category. Among them, 2 DEGs encoding chitin receptors (SECCE1Rv1G0028880.1, SECCE6Rv1G0388480.1), 4—L-type lectin domain receptor-like kinases (SECCE3Rv1G0157050.1, SECCE1Rv1G0007280.1, SECCE3Rv1G0160440.1, SECCE5Rv1G0367830.1), 5—cysteine-rich repeat (CRR) domain receptor-like kinases (SECCE2Rv1G0089280.1, SECCE3Rv1G0146170.1, SECCE2Rv1G0089270.1, SECCE7Rv1G0508800.1, SECCE2Rv1G0089300.1), 12—leucine-rich repeat (LRR) domain kinases (SECCE7Rv1G0458930.1, SECCE4Rv1G0275980.1, SECCE7Rv1G0499680.1, SECCE7Rv1G0515910.1, SECCE3Rv1G0149150.1, SECCE6Rv1G0419780.1, SECCEUnv1G0535240.1, SECCE4Rv1G0277950.1, SECCE1Rv1G0048800.1, SECCE3Rv1G0187650.1, SECCE7Rv1G0515830.1, SECCE2Rv1G0130680.1), 5—Wall-associated kinases (WAK) (SECCE4Rv1G0279050.1, SECCE3Rv1G0151000.1, SECCE4Rv1G0267570.1, SECCE6Rv1G0420150.1, SECCEUnv1G0531710.1), and 5—MAP kinases (SECCE3Rv1G0185330.1, SECCE3Rv1G0185340.1, SECCE4Rv1G0218820.1, SECCE5Rv1G0344410.1, SECCEUnv1G0542760.1) were upregulated. Besides, 4 upregulated DEGs encoding calmodulins (SECCE4Rv1G0259660.1, SECCE5Rv1G0352070.1, SECCE7Rv1G0481110.1, SECCEUnv1G0536530.1) and 2 upregulated Ca^2+^-influx glutamate receptors (SECCE5Rv1G0328480.1, SECCE5Rv1G0328470.1) and 7 upregulated DEGs related to the metabolism of inositol (SECCE7Rv1G0526300.1, SECCE7Rv1G0526290.1, SECCE4Rv1G0222660.1, SECCE7Rv1G0500690.1, SECCE5Rv1G0326360.1, SECCE4Rv1G0249470.1, SECCE7Rv1G0464430.1) were revealed, indicating that the activity of calcium and inositol signaling systems was presumably activated in the infected plants.

#### 2.1.6. The Transport-Related Category

93 DEGs (69 up- and 24 downregulated) were attributed to the transport-related category. The three largest groups of DEGs encoded ABC transporter-like proteins (18 upregulated DEGs), proteins involved in the transport of inorganic ions (21 up- and 6 downregulated DEGs), and proteins involved in the transport of non-photoassimilate organic compounds (14 up- and 10 downregulated DEGs). Based on the gene expression pattern it may be presumed that the transport of heavy metals, K^+^, and nitrates were enhanced during the infection. In addition, since 3 DEGs encoding invertases (SECCE6Rv1G0441380.1, SECCE5Rv1G0371670.1, SECCE2Rv1G0113540.1) and 4 DEGs encoding sugar transporters (SECCE2Rv1G0105290.1, SECCE4Rv1G0268710.1, SECCE7Rv1G0472490.1, SECCE1Rv1G0009970.1) were upregulated, it may be presumed that the transport of photoassimilates was increased following the infection.

### 2.2. Alterations in Lipase in Protease Activities, Terpenoid and Flavonoid Content in Rye Plants Following M. nivale Infection

To perform the physiological verification of our RNA-Seq data, we compared some physiological traits of control and *M. nivale*-infected plants that were hypothesized to be affected by the fungus colonization based on transcriptome profile. Our data revealed strong upregulation of protease-, lipase-, terpenoid-related genes as well as differential regulation of flavonoid-related genes. To check whether these enzymatic activities and terpenoid content were indeed increased following *M. nivale* infection and to assess the “output” of differential regulation of flavonoid-related genes in infected plants, protease, and lipase activities, as well as flavonoid and terpenoid contents, were determined.

**Figure 3:**
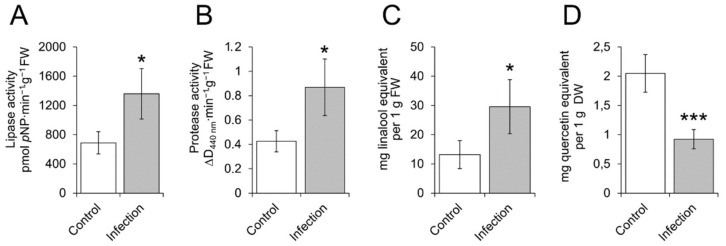
Lipase (**A**) and protease (**B**) activities as well as a terpenoid (**C**) and flavonoid content (**D**) in control and *Microdochium nivale*-infected rye plants (20th day post-inoculation). Asterisks (* or ***) show the significant differences (*p* < 0.05 or *p* < 0.001, respectively; one-tailed *t*-test). FW, fresh weight; DW, dry weight.

Protease and lipase activities were enhanced in rye plants infected with *M. nivale* compared to non-infected plants (Figure 3A,B). Terpenoid content was also increased in the *M. nivale*-infected plants compared to control plants (Figure 3C). In turn, free flavonoid content turned out to be significantly lower in infected plants than in control ones (Figure 3D).

### 2.3. The Effect of Exogenous Phytohormones on M. nivale Infection

**Figure 4:**
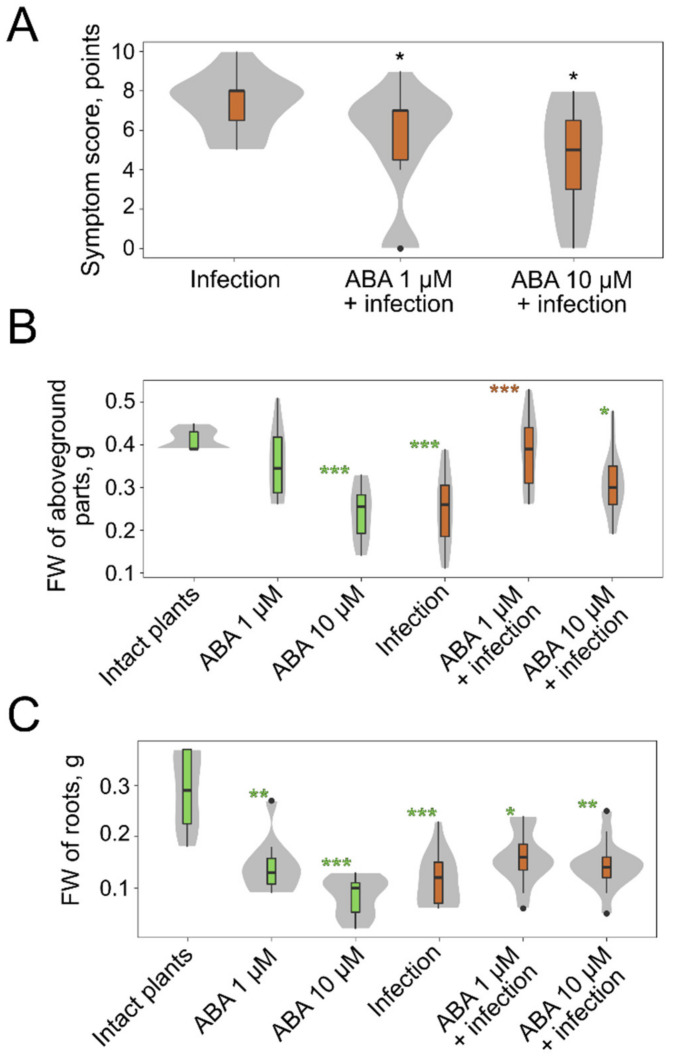
The effect of abscisic acid (ABA) pre-treatment on disease symptom development (necroses, desiccation) caused by *Microdochium nivale* in rye plants (**A**) and on the biomass (fresh weight) of the aboveground parts (**B**) and roots (**C**) of non-infected (green boxplots) and *M. nivale*-infected (brown boxplots) plants. Asterisks (*) in (**A**) show the significant differences (*p* < 0.05, Dunn’s test) between mock-pre-treated infected plants and ABA-pre-treated infected plants; non-infected plants had no symptoms (necroses, desiccation) after ABA treatment (symptom score = 0) (not shown in (**A**)). Green and brown asterisks in (**B**,**C**) show the significant difference between intact plants (non-pre-treated with ABA) and a given experimental group (green) and infected plants (non-pre-treated with ABA) and a given experimental group (brown): *—*p* <0.05; **—*p* < 0.01, ***—*p* < 0.001; Dunn’s test; *p*-values were adjusted using the Benjamini–Hochberg method. Grey areas behind the boxplots show the distributions of values; the widths of the grey areas correspond to the frequency of the values. FW—fresh weight.

Our RNA-Seq data revealed alterations in the expression of ABA-responsive genes during the infection that presumably pointed to a role of ABA in plant-*M. nivale* interactions. To check this hypothesis, the effect of exogenous ABA on the infection was assessed. The inoculation of plants with *M. nivale* resulted in the development of disease symptoms (necroses, desiccation of plant parts) and led to a decrease in the weight of both roots and aboveground plant parts (Figure 4A–C). ABA treatment (1 μM or 10 μM) reduced symptoms of disease (necroses, desiccation) in *M. nivale*-infected plants (Figure 4A). In addition, the weight of the aboveground plant parts was greater in infected plants pretreated with 1 μM of ABA than in infected plants pretreated with 10 μM of ABA, or non-pretreated with ABA (Figure 4B). Root weight reduced following *M. nivale* infection irrespective of whether the plants were pretreated with ABA or not (Figure 4C).

In non-infected plants, ABA did not cause any disease symptoms (necroses or desiccation) (symptom score = 0); but herewith, ABA at higher concentration (10 μM) reduced the weight of the aboveground plant parts and decreased root weight at both applied concentrations (1 and 10 μM) (Figure 4B,C). Thus, although ABA itself caused some growth retardation of rye plants, simultaneously, this phytohormone reduced disease symptoms and infection-related growth retardation of the aboveground plant parts. Therefore, ABA-induced responses likely contribute to the tolerance of rye plants to *M. nivale*.

**Figure 5:**
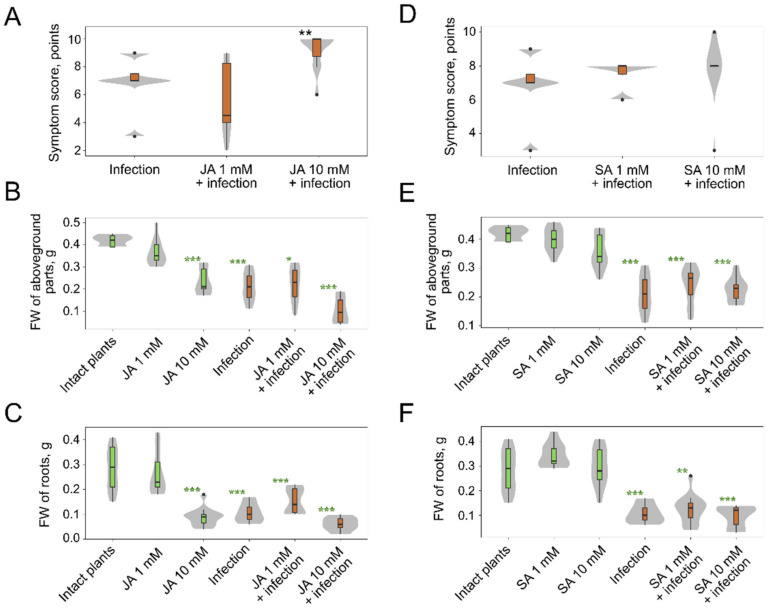
The effect of jasmonic acid (JA) (**A**–**C**) and salicylic acid (SA) (**D**–**F**) pre-treatment on disease symptom development (necroses, desiccation) caused by *Microdochium nivale* in rye plants (**A**,**D**) and on the biomass (fresh weight) of the aboveground parts (**B**,**E**) and roots (**C**,**F**) of non-infected (green boxplots) and *M. nivale*-infected (brown boxplots) plants. Asterisks (**) in **A** show the significant difference (*p* < 0.01, Dunn’s test) between mock-pre-treated infected plants and JA-pre-treated infected plants; non-infected plants had no symptoms (necroses, desiccation) after JA or SA treatment (symptom score = 0) (not shown in (**A**,**D**)). Green asterisks in (**B**,**C**,**E**,**F**) show the significant differences between control plants (non-pre-treated with JA/SA) and a given experimental group (green). *—*p* <0.05; **—*p* < 0.01, ***—*p* < 0.001; Dunn’s test; *p*-values were adjusted using the Benjamini-Hochberg method. Grey areas behind the boxplots show the distributions of values; the widths of the grey areas correspond to the frequency of the values.

Since JA or SA almost always play crucial roles in different plant-pathogen interactions [50,51,52], we also assessed the influence of JA and SA on the *M. nivale* infection despite that only rather small “fractions” of SA- or JA-regulated DEGs were revealed in our study. JA at higher concentration slightly exacerbated the manifestation of disease symptoms (Figure 5A) but simultaneously this JA concentration significantly reduced the weight of both roots and aboveground plant parts in non-infected plants. The lower JA concentration influenced the weight of neither control nor infected plants (Figure 5B,C). This means that JA exacerbates disease development only at the concentrations that reduce the fitness of non-infected plants. JA did not influence the weight of roots and aboveground parts in *M. nivale*-infected plants (Figure 5B,C). The SA treatment did not influence the disease score of infected plants and the weight of both infected and non-infected plants (Figure 5D–F).

## 3. Discussion

In the present study, alterations in the transcriptome profile of rye plants following *M. nivale* infection were characterized with the detailed functional classification of DEGs. In total, 1008 of genes were DEGs, 745 and 263 of which were up- and downregulated, respectively, in *M. nivale*-infected plants compared to control plants. Previous studies of transcriptome alterations in cereal plants following fungal infections revealed larger numbers of DEGs. For example, in *Lolium perenne* plants inoculated with *M. nivale*, 2354 genes were DEGs [30], and in rye plants, the expression of 3633 genes was altered following *Claviceps purpurea* infection [53]. Such a difference in the number of DEGs can be related to the fact that different infection stages might have been analyzed within particular studies. Herewith, at different stages, the plant transcriptional response to infection can be more (or less) pronounced. In addition, the interactions of plants and pathogens of different species can be associated with different numbers of regulated genes.

DEG classification allowed us to predict physiological reactions involved in rye resistance/susceptibility to *M. nivale*. For some of the DEG categories (“Protein degradation”, “Lipid degradation”, “Signalling”, “Flavonoid biosynthesis”, “Terpenoid biosynthesis”), the physiological verification of RNA-Seq data was performed by biochemical assays and treatment tests.

From the expression pattern of genes of the “Cell wall” category, it can be presumed that some intensification of lignin and cross-linking glycans (CLG) biosynthesis occurred during the infection that might promote the fortification of the plant cell wall. The upregulation of genes encoding particular transporters and invertases indicated that the transport of inorganic ions and photoassimilates might be enhanced in infected plants. Many stress-related genes were revealed among the infection-upregulated DEGs, including genes encoding xenobiotic detoxification-related proteins, PR proteins, ROS-related proteins, and receptors of elicitors. This indicates that the induction of the immune response occurred following the infection.

The remarkable modulation of expression in *M. nivale*-infected plants was observed for the genes of the “Secondary metabolism” category. The revealed upregulation of the phenylpropanoid biosynthesis-related genes was consistent with previous study showing the increase in the content of phenolic compounds following *M. nivale*-infection in triticale plants [11]. In particular, the phenylpropanoid pathway yields monolignols required for plant cell wall lignification, which impedes the disintegration of this compartment during phytopathogen attack. However, it has been shown that the pre-infection level of phenylpropanoids (phenolic compounds) was lower in more resistant to *M. nivale* cultivars of *Festulolium* plants than in more susceptible ones [22]. Therefore, the particular role of activation of the phenylpropanoid pathway in *M. nivale*-plant interactions remains to be determined.

In addition, many upregulated DEGs related to terpenoid synthesis were revealed. To carry out the physiological verification of these data, the terpenoid content was compared in control and *M. nivale*-infected plants. We showed that the level of terpenoids was increased following *M. nivale* infection. The increase in the expression of terpenoid-related genes as well as terpenoid content has been widely shown to take place in response to various biotic stressors [54,55,56,57,58,59,60]. Herewith, some infection-induced terpenoids act as phytoalexins [57,61]. Thus, the observed accumulation of terpenoids in response to *M. nivale* infection might restrain the extensive phytopathogen progression in planta.

In our study, genes related to flavonoid biosynthesis were found among both up- and downregulated DEGs. Therefore, it was difficult to judge whether flavonoid content was increased or decreased in *M. nivale*-infected plants compared to control plants. To check this, the level of free flavonoids was compared in control and infected plants. Free flavonoid content decreased in rye plants following *M. nivale* infection. It has been previously shown that flavonoids (especially catechin, proanthocyanidins, anthocyanidins) are often accumulated in plants infected with phytopathogenic fungi [62,63,64]. The role of flavonoids in plant-microbe interactions is often attributed to their antioxidant properties and direct antimicrobial activity [65]. Flavonoids can also act as scavengers of the plant cell wall degrading enzymes (e.g., xylanases, pectinases) secreted by fungal phytopathogens [65,66]. Therefore, flavonoids can be considered as the factors of plant resistance to pathogens. Our data showed that the concentration of flavonoids decreased following *M. nivale* infection, which was consistent with a high susceptibility of rye plants to this phytopathogen.

The observed decrease in flavonoid level can be related to the reduced synthesis of these compounds in the *M. nivale*-infected plants and/or to their degradation by *M. nivale*. It is unknown whether *M. nivale* can break down flavonoids; however, another phytopathogenic fungus *Sclerotinia sclerotiorum* has been shown to have such capacity [67]. In addition, the content of free flavonoids can decrease due to their incorporation into the plant cell wall. Flavonoids have been shown to bind callose, cellulose, and pectin in *Gossypium hirsutum* plants infected with *Xanthomonas campestris* [68]. Flavonoids can also participate in radical coupling reactions with monolignols acting as “non-conventional” lignin monomers facilitating plant cell wall fortification [69]. Our study shows that *M. nivale* infection affects the flavonoid content/distribution within the host plant. The elucidation of details and the particular consequences of *M. nivale*-caused alterations in flavonoid metabolism deserves further investigations in terms of the involvement of these diverse compounds in the potential restriction of *M. nivale*-caused disease.

The upregulation of genes related to the “Primary metabolism” category was the most pronounced during the *M. nivale* infection. Within this category, the “Protein metabolism” subcategory was the most enriched with DEGs, including many upregulated genes encoding protease-like proteins. The upregulation of genes for protease-like proteins was reflected in the proteolytic activity, which was greater in *M. nivale*-infected plants compared to the control ones. We cannot exclude that fungal proteases could contribute to the total pool of proteases within the infected plants. However, given that 24 plant genes encoding protease-like proteins were upregulated in infected plants, plant proteases were also evidently activated following *M. nivale* infection. Plant proteases are usually considered as the factors of resistance to pathogens. Herewith, plant proteases mediate the systemic acquired resistance (SAR) and priming, enhance perception of some pathogen effectors, and regulate the programmed cell death, which restricts pathogen spread from the infected tissue [70,71]. However, it should be noted that the programmed cell death can also facilitate disease development [29], and the breakdown of proteins may provide pathogens with available amino acids. Thus, given the pronounced activation of protein-degradation machinery in infected plants, the catabolism of proteins during *M. nivale*-caused disease deserves further in-depth investigations.

The enhanced catabolism of not only proteins but also lipids was evident in *M. nivale* infected plants. Many plant lipase-encoding genes were upregulated and the lipase activity increased following the *M. nivale* infection. Similar to the protease activity, we cannot rule out that fungal lipases contributed to the total lipase activity within the infected plants. The role of lipid degradation in plant-microbe interactions is often associated with oxylipin biosynthesis. Fatty acids released from lipid esters are converted in the lipoxygenase pathway yielding various oxylipins, such as fatty acid hydroperoxides, divinyl ethers, oxo-acids, and aldehydes, allene oxides, and cyclopentenones such as jasmonic acid, which participate in many physiological processes including plant-phytopathogen interactions [72,73,74]. However, in our study, we did not observe the significant upregulation of genes related to oxylipin biosynthesis in the *M. nivale*-infected plants despite that lipid degradation was strongly pronounced.

The lipid metabolism seems to be a crucial aspect of *M. nivale* physiology. Being a psychrotolerant fungus, *M. nivale* is capable of causing diseases, not only at moderate, but also at low temperatures. Herewith, in contrast to the other snow mold-causing phytopathogens (such as psychrophilic *Typhula ishikariensis*), *M. nivale* does not produce antifreeze proteins [75]. The psychrotolerance of *M. nivale* is considered to be a result of the alterations in lipid metabolism: (1) the unsaturated lipids as well as neutral triacylglycerols are accumulated in *M. nivale* in response to low temperatures [76,77], (2) the *M. nivale* extracellular lipase remains active at low temperatures [78]. Therefore, the consumption of lipid precursors can be important for *M. nivale* fitness. In turn, phytopathogens can manipulate the host’s enzymatic machineries to facilitate the formation of a growth substrate [29]. Therefore, we presume that lipids are one of the crucial “targets” for *M. nivale* within the host plants and that the upregulation of plant lipase-encoding genes represents a susceptible response of rye plants to *M. nivale* infection, which “forces” the host to supply the pathogen with necessary metabolites.

Most (if not all) plant infectious diseases are associated with phytohormone perturbations. We found that some ABA-regulated genes were differentially expressed during the infection, indicating that ABA might play a role in plant-*M. nivale* interaction. To check this, we compared the progression of *M. nivale* infection in non-pretreated and ABA-pretreated plants. The infection symptoms were less pronounced in plants pretreated with ABA. Thus, our results show that ABA contributes to plant resistance/tolerance to *M. nivale*. In general, ABA plays a dual role in plant-pathogen interactions. On the one hand, ABA can contribute to the resistance/tolerance of plants to some pathogens [64,79,80]. On the other hand, ABA can reduce host resistance to pathogens. Herewith, ABA has been shown to suppress SA and JA-mediated immune responses [29,81].

We also assessed the influence of SA and JA on the *M. nivale*-caused disease development since these two phytohormones are considered crucial regulators of most (if not all) plant-pathogen interactions. Herewith, both JA and SA can contribute to either plant susceptibility or resistance depending on several factors such as the pathogen’s lifestyle [50,82]. Unexpectedly, neither JA nor SA affected the disease development caused by *M. nivale* in rye plants. To the best of our knowledge, the cases, in which both JA and SA did not influence the disease progression, have not been described to date for any plant-pathogen interactions. This likely means that *M. nivale* interactions are controlled in a manner non-conventional for most of the plant-pathogen interactions.

## 4. Methods

### 4.1. Plant and Fungi Growth Conditions and Plant Inoculation

*Microdochium nivale* strain F00608 from the collection of the Laboratory of Plant Infectious Diseases FRC KazSC RAS (Kazan, Russia) and winter rye (*Secale cereale* L.) cultivar Ogonek from Tatar Scientific Research Institute of Agriculture FRC KazSC RAS (Kazan, Russia) were used in this study. Seeds were washed and sterilized as described previously [32]. Seeds were germinated for 2 days at 25 °C in dark. Seedlings were transferred to individual sterile 50 mL glass tubes with 7 mL of 1/4 diluted Murashige and Skoog medium without organic carbon. A highly virulent strain *M. nivale* F00608 was grown on potato sucrose agar (PSA) at 20 °C in dark for 10 days.

To obtain inoculum, agar plugs of 8 mm diameter were cut from the marginal zone of the fungal colony. Each seedling was inoculated with one agar plug containing the mycelium of *M. nivale*. Mycelium-free agar plugs were used for mock-treatment of the control plants. Inoculated plants were cultured for 20 days at 20 °C with 16/8 h photoperiod (day/night).

### 4.2. The Evaluation of the Symptom Manifestation

**Figure 6:**
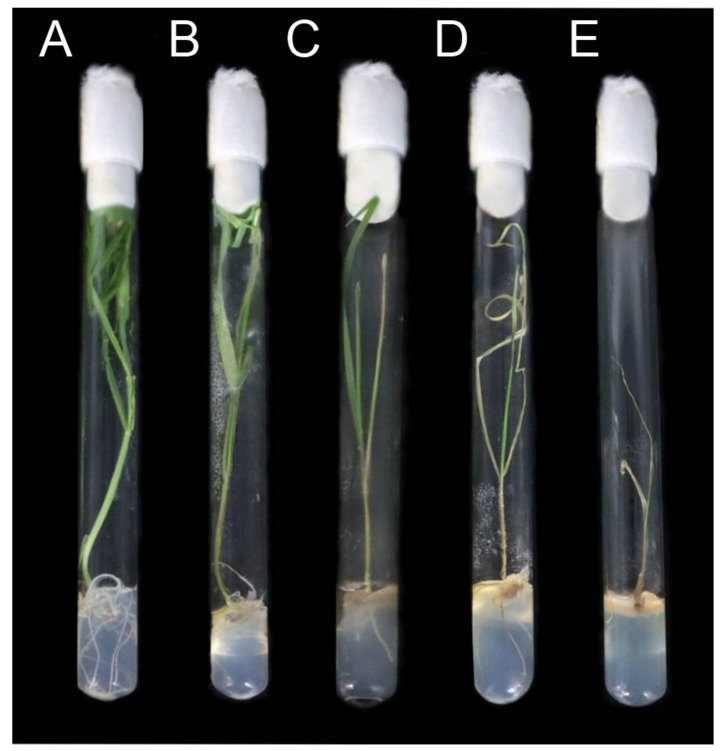
The example of symptom score estimation for the *Microdochium nivale*-infected rye plants. (**A**) symptomless plant (symptom score = 0). (**B**) Light symptoms (symptom score = 2). (**C**) Moderate symptoms (symptom score = 5). (**D**) Heavy symptoms (symptom score = 8). (**E**) Maximal symptom severity (symptom score = 10).

The infection symptom development was scored on the discrete eleven-point scale (0—the infection was not manifested, 10—the infection was manifested strongly) (Figure 6). Symptom score was determined by visual assessment of necrosis and desiccation of the aboveground parts of a plant. The fresh weight of aboveground plant parts and roots of intact and infected plants was measured and compared. The described metrics were also collected for the infected and non-infected rye plants treated with exogenous abscisic acid (ABA) (Sigma-Aldrich, St. Louis, MO, USA), methyl ether of jasmonic acid (JA) (Sigma-Aldrich, St. Louis, MO, USA), or sodium salt of salicylic acid (SA) (Sigma-Aldrich, St. Louis, MO, USA) solutions to reveal the possible effect of these phytohormones on the infection.

### 4.3. Treatment with Exogenous Phytohormones

Two-leaf-stage rye seedlings grown in sterile 50 mL glass tubes were sprayed with 250 µL of SA (1 mM or 10 mM), or JA (1 mM or 10 mM), or ABA (1 µM or 10 µM). The phytohormone solutions were sterilized by filtration through 0.2 µM pore sterile filters (Corning, Berlin, Germany). Distilled water was used for the control treatment. Phytohormone- and water-treated plants were incubated for 1 day and then inoculated with agar plugs (with or without fungal mycelium) as described above.

### 4.4. Estimation of Total Terpenoid Content

Total terpenoid content was estimated as described earlier [83]. Briefly, 500 mg of fresh plant material (leaf sheaths taken from control plants and *M. nivale*-infected plants with a disease score of 5 points) were homogenized in ice-cold mortar in 3.5 mL of 96% methanol. The obtained mixture was gently vortexed and incubated for 48 h in a dark place at room temperature. The extracts were centrifuged at 5000× *g* for 10 min and the supernatants were collected. Then, 200 µL of extracts were mixed with 1.5 mL of chloroform, vortexed, and incubated for 3 min. Hundred µL of concentrated sulfuric acid were added to each sample. The samples were incubated for 2 h in a dark place until a brown precipitate formed. The supernatants were removed. The sample precipitates were resuspended in 500 µL of 96% methanol. The absorption of samples was measured at 538 nm using РВ2201В spectrophotometer (SOLAR, Minsk, Belarus); 96% methanol was used as a blank solution. Linalool (Sigma-Aldrich, St. Louis, MO, USA) was used for standard curve calculation.

### 4.5. Estimation of Total Flavonoid Content

Total flavonoid content in plants was assessed using the previously described method [84,85] with some modifications. Briefly, 50 mg of dry plant biomass (leaf sheaths taken from control plants and *M. nivale*-infected plants with a disease score of 5 points) were homogenized in mortar in 1 mL of 96% methanol. The obtained mixture was gently vortexed and incubated for 40 min at 45 °C. The extracts were centrifuged at 5000× *g* for 10 min, and the supernatants were collected. For colorimetric reaction, 250 µL of 2% AlCl_3_ in 96% methanol was added to 250 µL of the obtained extracts. Samples were incubated at room temperature in dark for 60 min. The absorption of samples was measured at 430 nm using РВ2201В spectrophotometer (SOLAR, Minsk, Belarus); 96% methanol was used as a blank solution. Quercetin (Sigma-Aldrich, St. Louis, MO, USA) solutions in methanol were used for standard curve calculation.

### 4.6. Lipolytic and Proteolytic Activity Measurement

To determine the levels of lipolytic and proteolytic activities, 100 mg of plant material (leaf sheaths taken from control plants and *M. nivale*-infected plants with disease score of 5 points) were ground in 1 mL of cold Tris-HCl buffer 100 mM, pH 7.5 in mortars. The homogenates were centrifuged (7000× *g*, 10 min), and the supernatants were taken for the enzymatic activity measurement.

To assess the proteolytic activity, 125 μL of 100 mM Tris-HCl buffer (pH 7.5) and 125 μL of 1.5% azocasein (Sigma-Aldrich, St. Louis, MO, USA) in deionized water were mixed with 100 μL of the cultural supernatant and incubated 60 min at 37 °C. The reactions were stopped by the addition of 100 μL of 10% trichloroacetic acid (Sigma-Aldrich, St. Louis, MO, USA). The sediment was removed by centrifugation (5000× *g*, 10 min, 25 °C). Four hundred μL of the supernatant and 133 μL of 1.0 N NaOH were mixed, then the absorbance at 440 nm was measured using PB2201B spectrophotometer (SOLAR, Minsk, Belarus). One unit of protease activity was defined as the amount of enzyme required to produce an absorbance change of 1.0 per min per 1 g of fresh weight [86].

To assess the lipolytic activity, 450 μL of 100 mM Tris-HCl buffer and 30 μL of the substrate solution (100 mM *p*-nitrophenyl butyrate, *p*NPB, in 2-propanol) were mixed with 100 μL of the supernatant and incubated for 15 min at 37 °C. The test samples were cooled on ice and centrifuged (2000× *g*, 5 min, 4 °C). The absorbance of the released *p*-nitrophenol (*p*NP) was measured at 410 nm using PB2201B spectrophotometer (SOLAR, Minsk, Belarus). The lipolytic activity was defined as pmol *p*NP per min per 1 g of fresh weight [87].

### 4.7. RNA Isolation, Sequencing

Plant material (leaf sheaths taken from control plants and *M. nivale*-infected plants with a disease score of 5 points) was ground in liquid nitrogen. The obtained powder was resuspended in 1 mL of ExtractRNA Reagent (Evrogen, Moscow, Russia). The subsequent procedures were performed according to the manufacturer’s instructions. Residual DNA was eliminated using a DNA-free kit (Life Technologies, Carlsbad, CA, USA). RNA quantity and quality were analyzed using a Qubit fluorimeter (Life Technologies, Carlsbad, CA, USA) and Qsep100 DNA Analyzer (Bioptic, New Taipei City, Taiwan), respectively.

For RNA-Seq, total RNA (1 μg) was enriched with mRNA using NEBNext Poly(A) mRNA Magnetic Isolation Module (New England Biolabs, Hitchin, UK). mRNA was processed using NEBNext Ultra II Directional RNA Library Prep Kit for Illumina (New England Biolabs, Hitchin, UK) according to the manufacturer’s instruction. The quality and quantity of the cDNA libraries before sequencing were monitored using an Agilent 2100 Bioanalyzer (Agilent, Santa Clara, CA, USA) and a CFX96 Touch Real-Time PCR Detection System (Bio-Rad, Hercules, CA, USA). For the quantification of the cDNA libraries, the EVA Green I PCR-Kit (Synthol, Moscow, Russia) and primers for Illumina adapters (Evrogen, Moscow, Russia) were used; PhiX Control (Illumina, San Diego, CA, USA) was used as a concentration standard. Libraries were sequenced in three biological replicates. Sequencing was conducted on Illumina HiSeq 2500 (Illumina, San Diego, CA, USA) at the Joint KFU–Riken Laboratory, Kazan Federal University (Kazan, Russia).

### 4.8. The Analysis of Differentially Expressed Genes

Raw reads generated in this study are available at the NCBI BioProject under the accession number PRJNA785089. The quality of the reads was assessed using FastQC (http://www.bioinformatics.babraham.ac.uk/projects/fastqc/, accessed on 13 November 2021). Reads with a q-score < 30 and rRNA-corresponding reads were filtered out using Trimmomatic and SortMeRNA, respectively [88,89]. Pseudo-alignment and quantification of filtered reads were carried out using Kallisto [90] with default parameters and reference transcript sequences of *Secale cereale* inbred line ‘Lo7′ [33]. The edgeR package [91] was used to reveal differentially expressed genes (DEGs). Genes that had TMM-normalized read counts per million (CPM) values ≥ 1 in all replicates within at least one of the experimental conditions were considered to be expressed in our study. Genes with |log_2_FC| < 1 and FDR < 0.05 were considered to be DEGs. To interpret the RNA-Seq data in terms of general physiology, the reference rye transcript annotations were fused to our RNA-Seq data. Then, all transcripts were additionally annotated using eggNOG mapper [92] with default parameters, and BLAST+ against the reference proteomes of *Arabidopsis thaliana*, *Oryza sativa*, *Triticum aestivum*, and *Hordeum vulgare* obtained from the UniProt website (http://uniprot.org/, accessed on the 22 November 2021). The GO, KEGG, KOG, and CAZy mappings obtained from eggNOG were used to perform the automatic classification of DEGs into functional categories using R software. The merged classification based on the above-mentioned databases was created, manually checked, edited, and enriched by the “missed” genes based on the information from the UniProt database.

The functional DEG classification was hierarchically organized into 4 levels of categories. Categories of successive levels are nested, with the 1st level categories being the largest and the 4th level categories being the smallest. The number of up- and downregulated DEGs in each category were calculated. The classification markup and DEG numbers are given in Supplementary Table S2. The measures of the expression levels are given in Supplementary Table S3.

## 5. Conclusions and Future Perspectives

The first study on the transcriptome response of a crop plant (rye) to *M. nivale* infection was carried out. The detailed functional classification of differentially expressed genes (DEGs) allowed us to predict plant physiological reactions associated with the development of the disease caused by this pathogen. Some of the predicted reactions were physiologically verified and investigated or discussed in terms of their involvement in plant resistance/tolerance or susceptibility to *M. nivale*.

ABA significantly contributes to plant’s resistance/tolerance to *M. nivale*. The expression of some ABA-regulated genes changes following the infection, while pre-infectional ABA treatment reduces symptom manifestation. Flavonoids were also presumed to confer the resistance/tolerance to *M. nivale* since an extensive symptom manifestation was associated with the decrease in the content of these compounds, many of which have been previously shown to act as the suppressors of different plant diseases [65].

The studied infection is also associated with the increased levels of terpenoids and proteolytic activity (as well as upregulation of corresponding genes in plants). Terpenoids and proteases can be presumed to determine both the resistance and susceptibility of plants to *M. nivale*, and further experiments are required to specify the particular consequences of the increased terpenoid level and enhanced proteolytic activity for plant-*M. nivale* interactions.

The upregulation of plant genes encoding lipases and the induction of lipolytic activity in *M. nivale*-infected plants revealed in our study were presumed to play an important role in plant susceptibility to the studied phytopathogen. Lipid metabolism has been previously shown to have particular meaning for the ecological fitness of *M. nivale* [76,77]; herewith, the necessary compounds are often obtained by different pathogens via manipulating the host plant enzymatic machineries in the frameworks of susceptible host responses [29]. Therefore, we hypothesize that *M. nivale* exploits its host plants to supply itself with lipid precursors. The identification of genetic bases of such exploitation is important not only for an understanding of fundamental aspects of plant-*M. nivale* interactions but also for obtaining resistant/tolerant cultivars using genome editing techniques.

Our work disclosed important aspects of plant-*M. nivale* interactions and outlined the directions for future studies on poorly characterized plant diseases caused by this devastating phytopathogen. To obtain a more general picture of *M. nivale* infection and to get a deeper insight into physiological determinants of plant resistance and susceptibility to this phytopathogen, the additional transcriptome profiling coupled with physiological verification should be performed for different stages of the disease, various environmental conditions, cultivars with different level of susceptibility, and different organs, including roots that have been previously shown to suffer even more strongly from the *M. nivale* infection than the aboveground plant parts [32].

## Data Availability

Raw reads generated in this study are available at the NCBI BioProject under the accession number PRJNA785089.

## Supplementary Materials

The following are available online at https://www.mdpi.com/article/10.3390/plants10122723/s1, Table S1: the details of RNA-Seq and read mapping; Table S2: gene functional category markup and several differentially expressed genes in each category; Table S3: differential gene expression in each functional category.
